# Hypertensive heart disease: risk factors, complications and mechanisms

**DOI:** 10.3389/fcvm.2023.1205475

**Published:** 2023-06-05

**Authors:** Sepiso K. Masenga, Annet Kirabo

**Affiliations:** ^1^ HAND Research Group, School of Medicine and Health Sciences, Mulungushi University, Livingstone Cam-Pus, Livingstone, Zambia; ^2^ School of Medicine, University of Zambia, Lusaka, Zambia; ^3^ Department of Medicine, Vanderbilt University Medical Centre, Nashville, TN, United States

**Keywords:** hypertensive heart disease, heart failure, salt, hypertension, coronary artery disease, atrial fibrillation, immunity

## Abstract

Hypertensive heart disease constitutes functional and structural dysfunction and pathogenesis occurring primarily in the left ventricle, the left atrium and the coronary arteries due to chronic uncontrolled hypertension. Hypertensive heart disease is underreported and the mechanisms underlying its correlates and complications are not well elaborated. In this review, we summarize the current understanding of hypertensive heart disease, we discuss in detail the mechanisms associated with development and complications of hypertensive heart disease especially left ventricular hypertrophy, atrial fibrillation, heart failure and coronary artery disease. We also briefly highlight the role of dietary salt, immunity and genetic predisposition in hypertensive heart disease pathogenesis.

## Introduction

Hypertensive heart disease is a term applied to abnormalities of the heart, involving structure and function of the left ventricle, the left atrium and intramural coronary arteries due to sustained elevated blood pressure (1). Although the blood pressure cut-off criteria for the diagnosis of hypertension differs based on the American (2) and European (3, 4) guidelines, most of the recommendations are similar (5). Moreover, the complications of chronic hypertension remain the same. Consensus on the criteria for hypertensive heart disease is not yet universal. However the European criteria as proposed by Gonzalez-Maqueda et al. from the Spanish Society of Cardiology define and classify hypertensive heart disease based on the acronym “VIA” referring to alterations of function and structure occurring in the left ventricle (V), myocardial ischaemia (I), and atrial fibrillation (6). In general, both the European and American hypertension guidelines or other international working groups/societies agree that hypertensive heart disease may involve left ventricular hypertrophy (LVH), left atrial dilatation, systolic and diastolic dysfunction including some clinical symptoms or manifestations such as arrhythmias, myocardial ischemia and heart failure (1, 6). LVH is one of the earliest manifestations of hypertensive heart disease and is thought to be a compensatory mechanism to minimize the increase in ventricular wall stress and an intermediate pathological change in the advancement of hypertensive heart disease (7). However, LVH may progress to complications such as heart failure, arrhythmias, sudden cardiac arrest, ischemic stroke, end stage renal disease (ESRD) and death (8–10).

Globally, the age-standardized prevalence of hypertension in women and in men was 32% and 34%, respectively (11). The prevalence has been increasing with time (12), by more than 138% between 1990 and 2019, affecting a total of 20 million (13). Among persons with hypertension, LVH prevalence is about 40% (14) and black persons tend to have increased left ventricular mass and more severe diastolic dysfunction compared to white persons (1, 15, 16).

## Risk factors of hypertensive heart disease

**Figure 1:**
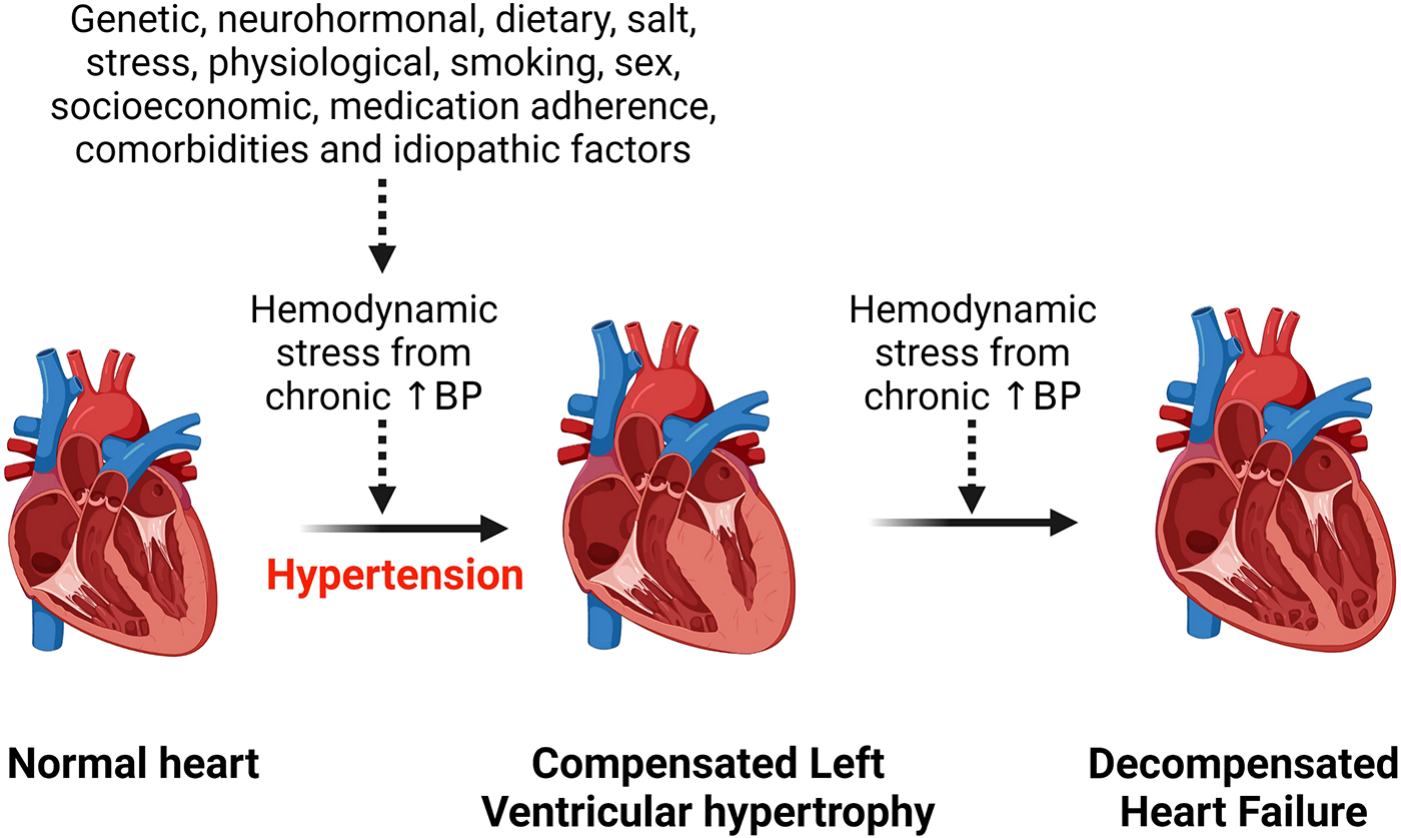
Effect of chronic hemodynamic stress on the heart. Chronic hypertension resulting from various stimuli leads to left ventricular hypertrophy as a compensation for the hemodynamic stress and metabolic demand on the heart. However, heart failure results when the heart can no longer withstand the persistent hemodynamic stress. BP, blood pressure.

Hypertension is the most common risk factor for development of hypertensive heart disease (1). Additional risk factors include older age, ethnicity, being overweight, physical inactivity, excess dietary salt intake, smoking, alcohol intake, concomitant diseases such as diabetes mellitus (17–19). All these factors contribute to an increased hemodynamic stress on the heart and with chronicity, the left ventricle of the heart hypertrophies to compensate for the load, but in the long run this can lead to heart failure (Figure 1). Obesity is an important risk factor for the development of hypertensive heart disease due to the associated increase in renin secretion that is mediated by leptin production via adipose cells, resulting in blood pressure elevation and exacerbating already existing hypertension (20). Moreover, obesity-related increase in sympathetic tone counteracts the body's compensatory mechanisms for the abnormally elevated renin and aldosterone level that induce cardiac fibrosis and endothelial dysfunction and contribute to the development and complications of hypertensive heart disease (20, 21). Beside altering hemodynamics, obesity also contributes to hypertensive heart disease by inducing inflammation, lipid accumulation in tissue and dysregulating several intracellular pathways (22–24). Obesity and hypertension synergize through overlapping neurohormonal pathways and contribute to hypertensive heart disease and its complications such as LVH and heart failure (25). However, the mechanisms are still complex.

## Mechanisms of hypertensive heart disease and its complications

The common complication of hypertensive heart disease is either diastolic heart failure, systolic failure, or a combination of the two and individuals with hypertensive heart disease have a higher risk for development of atrial fibrillation (AFib), perioperative ischaemia, coronary artery disease, rehospitalization, kidney disease, heart valve diseases, aortic dissection, intramural hematoma, and aortic ulcer (26–28).

### Left ventricular hypertrophy mechanisms in hypertensive heart disease

LVH is an early compensatory response to hemodynamic overload in chronic hypertension (29, 30). LVH can predispose to cardiovascular events in individuals with hypertension who have coronary artery calcifications but without symptoms (31) or in individuals with hypertension independent of treatment and other existing cardiovascular risk factors (32). In addition, malignant LVH characterized by elevated biomarkers of cardiac injury and hemodynamic stress such as N-terminal prohormone of brain natriuretic peptide (NT-proBNP) and troponins is associated with severe adverse outcomes such as heart failure with reduced ejection fraction, left ventricular dysfunction and cardiovascular death (33). Thus, high-sensitive cardiac troponin T (hs-cTnT) and NT-proBNP levels can be used to identify patients who are more likely to develop adverse outcomes (34). It is also important to note that malignant LVH and related adverse events may be more pronounced in black persons compared to white persons (35). However, intensive therapy to lower blood pressure can prevent malignant LVH and reduce the risk for adverse events (36). The pathogenesis and severity of LVH is different by sex. For example, aortic characteristic impedance, systemic vascular resistance, augmentation index, and carotid-femoral pulse wave velocity and proximal aortic compliance are independently associated with relative wall thickness in women but not in men (37). LVH is therefore, more often and more pronounced in women compared to men (38, 39). LVH can be pathological and physiological in pattern (40, 41). The distinguishing pathological changes include increased extracellular connective tissue relative to myocytes without commensurate capillary growth, and myocardial fibrosis that often manifests as diastolic dysfunction (1, 30, 42). In Physiological LVH, extracellular matrix and micro-vessel increase is proportional to the myocyte hypertrophy without deleterious effects on left ventricular function (42). This physiological pattern is seen as a normal response to physical exercise (42).

The mechanisms by which chronic hypertension leads to LVH may involve gap junction lateralization and overexpression of the fatty acid transporter cluster of differentiation 36 (CD36), redox-sensitive Protein Kinase C (PKC), increased oxidative stress, increased matrix metalloproteinase-2 (MMP-2) activity, abnormal Ca^2+^ homeostasis, increased activation of the phosphoinositide 3-kinase (PI3K)/protein kinase B (AKT) pathway, apoptosis, and abnormal regulation of junctional proteins (7, 43). All these compensatory changes participate in an orchestrated manner to compensate for increased stress, metabolic and functional demand but are likely to also induce decompensated heart failure when the pathological insult is persistent (7, 44, 45). At cellular level, LVH is a consequence of an increase in cardiac myocyte size due to functional demand but chronic blood pressure elevations may lead to death of cardiomyocytes progressing to dilated cardiomyopathy, a feature described as a transition from compensated to decompensated heart failure and mediated by neurohormonal signaling (45). The whole mark and characteristic feature of LVH is an increase in cardiomyocyte with fibrotic changes including medial hypertrophy and perivascular fibrosis (1). The structural features of LVH can either take concentric or an eccentric pattern depending on volume load, genetic factors, specific alterations of the extracellular matrix, neurohormonal milieu, pressure load severity, duration, rapidity of onset, concomitant medical conditions such as cardiovascular disease, metabolic disease such as diabetes mellitus and demographic factors such as age, race/ethnicity, gender (1, 46). The ultimate consequence of LVH with continued hemodynamic stress is progression to heart failure with either a preserved or a reduced left ventricular ejection fraction (47, 48). There is evidence of LVH regression with use of different classes of antihypertensive medication, however, there is considerable variability in individual responses including sex differences (49) owing to variability of pathological changes per individual and a number of studies have reported failure to regress LVH even when blood pressure is controlled (49–54).

**Figure 2:**
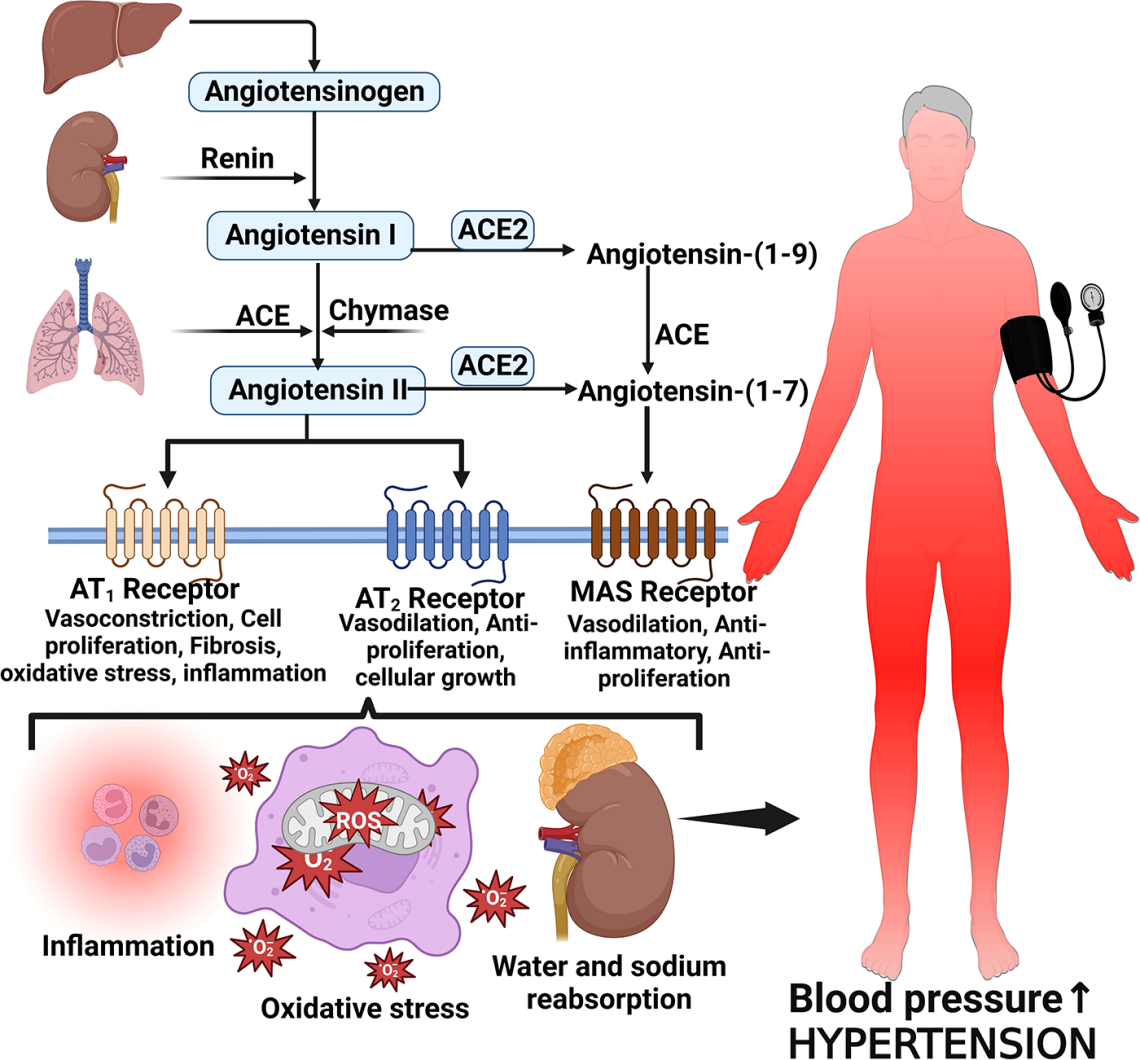
Angiotensin II formation and effects on blood pressure. Angiotensinogen is produced by the liver and converted to angiotensin I by the action of renin produced by the juxtaglomerular cells of the kidney. Angiotensin I is then converted to angiotensin II via angiotensin-converting enzyme (ACE). Angiotensin II binds to its receptors to induce several activities such as inflammation, vasoconstriction oxidative stress and reabsorption of water and sodium leading to elevated blood pressure and hypertension; MAS, marker assisted selected.

Several proteins, mediators and cellular responses such as angiotensin II, cardiac myosin-binding protein C, endothelin-1, S-thiolated protein, norepinephrine, Rho and Ras proteins, thiols, oxidative stress, heat shock proteins, Fractalkine/C-X3-C Motif Chemokine Ligand 1 (CX3CL1), leukotriene-A4 hydrolase, calcineurin, and some kinases have been implicated in LVH which is associated with chronic elevated blood pressure (42, 55–57). The enzymatic cleavage of angiotensinogen by renin converts angiotensinogen to Angiotensin I and then angiotensin converting enzyme (ACE) converts Angiotensin I to Angiotensin II (58, 59). Angiotensin II is the main effector molecule of the renin angiotensin aldosterone system (RAAS) that serves to control blood pressure (58). Angiotensin II increases blood pressure by inducing water and sodium reabsorption, inducing vasoconstriction and exerting proliferative, pro-inflammatory and pro-fibrotic activities by binding to angiotensin type 1 and 2 receptors (59, 60) (Figure 2).

Angiotensin II increases blood pressure and induces pathological features characteristic of hypertensive heart disease by activating angiotensin II receptors, regulating cardiac contractility, cardiac remodeling, growth, inflammation, apoptosis and impulse propagation (59, 61). Angiotensin II activates extracellular signal-regulated kinase (ERK)/ mitogen-activated protein kinases (MAPKs) pathways via G protein-coupled receptors (GPCRs) or growth factor-stimulated tyrosine kinase receptors leading to an increase in protein synthesis, extracellular matrix proteins and activation of endothelin-1 effects which result in induction of cardiac fibrosis (42). Heat shock proteins 90 (HSP90) mediate cardiac hypertrophy that is induced by angiotensin II through the stabilization of IкB kinase (IKK) complex (62). Activation of the Nuclear factor kappa-light-chain-enhancer of activated B cells (NF-*κ*B) during hemodynamic stress, inflammation and reactive oxygen species (ROS) production in cardiac myocytes also contributes to cardiac hypertrophy in hypertensive heart disease (42).

Another mechanism associated with LVH is activation of calcineurin and calmodulin kinase II (CaMKII) due to enhanced sensitivity to calcium resulting in calcineurin binding to and dephosphorylating nuclear factor of activated T cells (NFAT)(42, 63). This step increases hypertrophic gene expression through mechanisms that are not yet clear (63). Intergrins have also been implicated in LVH mediated by RAAS and MAPKs pathways (42).

### Mechanisms of heart failure associated with hypertensive heart disease

LVH progresses to heart failure when the compensatory mechanisms have failed to meet the functional and metabolic demands of the myocardium (64). Heart failure is a progressive clinical syndrome characterized by reduced ability of the heart to pump blood to meet the body's metabolic demand (65, 66). Symptoms include dyspnoea, fatigue, peripheral oedema or distended jugular veins (65, 66). The symptoms/signs are caused by abnormality in cardiac function and structure and the clinical syndrome is characterized by an increase in natriuretic peptide levels and pulmonary or systemic congestion (67). Heart failure can either be acute or chronic (68). The classification of heart failure is based on left ventricular ejection fraction (LVEF) and can present either with reduced ejection fraction (HFrEF)) or preserved ejection fraction (HFpEF) (65, 67). The clinical stages of heart failure based on United states (US) guidelines fall into four categories (67, 69) as shown in Table 1. The pathogenesis, risk factors and therapeutic response in heart failure is sex dependent. For example, women are more susceptible to traditional risk factors for heart failure and have more severe symptoms especially with higher left ventricular ejection fraction compared to men (70, 71). However, in terms of the adverse outcomes such as hospitalization and mortality, the prognosis regardless of the ejection fraction state, appears to be better for women than men (72). In general, specific data on sex differences in heart failure is still limited due to the fact that women are underrepresented in most studies (72). Also, while women may have more disease severity on some outcomes, this is not the case with other outcomes or symptoms such as plaque rupture which is more common in men (71, 73).

**Table 1:** Clinical stages of heart failure based on symptoms according to AHA/ACC/HFSA guidelines.

Stage	Description
A	Patients at risk for heart failure HF but without current or prior symptoms or signs of heart failure and without structural or biomarker evidence of heart disease.
B	Pre-heart failure stage for patients without current or prior symptoms or signs of heart failure but have evidence of structural heart disease or abnormal cardiac function, or elevated natriuretic peptide levels.
C	For patients with current or prior symptoms and/or signs of heart failure caused by a structural and/or functional cardiac abnormality.
D	Advanced heart failure; for patients with severe symptoms and/or signs of HF at rest, recurrent hospitalizations despite guideline-directed medical therapy (GDMT), refractory or intolerant to GDMT, requiring advanced therapies such as consideration for transplant, mechanical circulatory support, or palliative care.

ACC, American college of cardiology; AHA, American heart association; HFSA, heart failure society of America.

The transition from hypertrophy to heart failure in hypertensive heart disease is driven by several cellular mediators many of which are progressive changes already explained under LVH and these include oxidant stress, apoptosis, insufficient angiogenesis, mitochondria dysfunction, metabolic derangements and fetal gene program induction (74, 75). The underlying pathways and cellular mediators that are activated to mediate the pathology of heart failure in hypertensive heart disease include peroxisome proliferator-activated receptor-*γ* coactivator-1α (PGC-1α) and PGC-1β (64), GPCRs, p38, ERK1/2, JNK, CAMKII, protein kinases (type C, G, and A), Growth factor-mediated stimulation of mechanistic target of rapamycin (mTOR) (74), epigenetic modulators (such as NFAT, MEF2) and transient receptor potentials (74, 76). Most of these pathways have been reviewed elsewhere (74). PGC-1*α* and PGC-1β both play a role in the maintenance of cardiac function during pressure overload such that in the progression to heart failure, a deficiency of PGC-1β is shown to accelerate the transition (64). The hormone ligands that mediate the activation of these pathways include angiotensin II, endothelin 1, *α*-adrenergic receptors and *β*- adrenergic receptors (74). At organ level, multiple cardiac, vascular, and non-cardiac abnormalities associated with hypertensive heart disease underly the pathophysiology of heart failure (77, 78). These include impaired structural and functional changes of the left ventricle, myocardial ischemia, autonomic deregulation, endothelial dysfunction and vascular stiffening (77, 79). The RAAS is an important contributor that plays a central role in the transition from LVH to heart failure in hypertensive heart disease and has been explained above.

### Mechanisms of conduction arrhythmias associated with hypertensive heart disease

**Figure 3:**
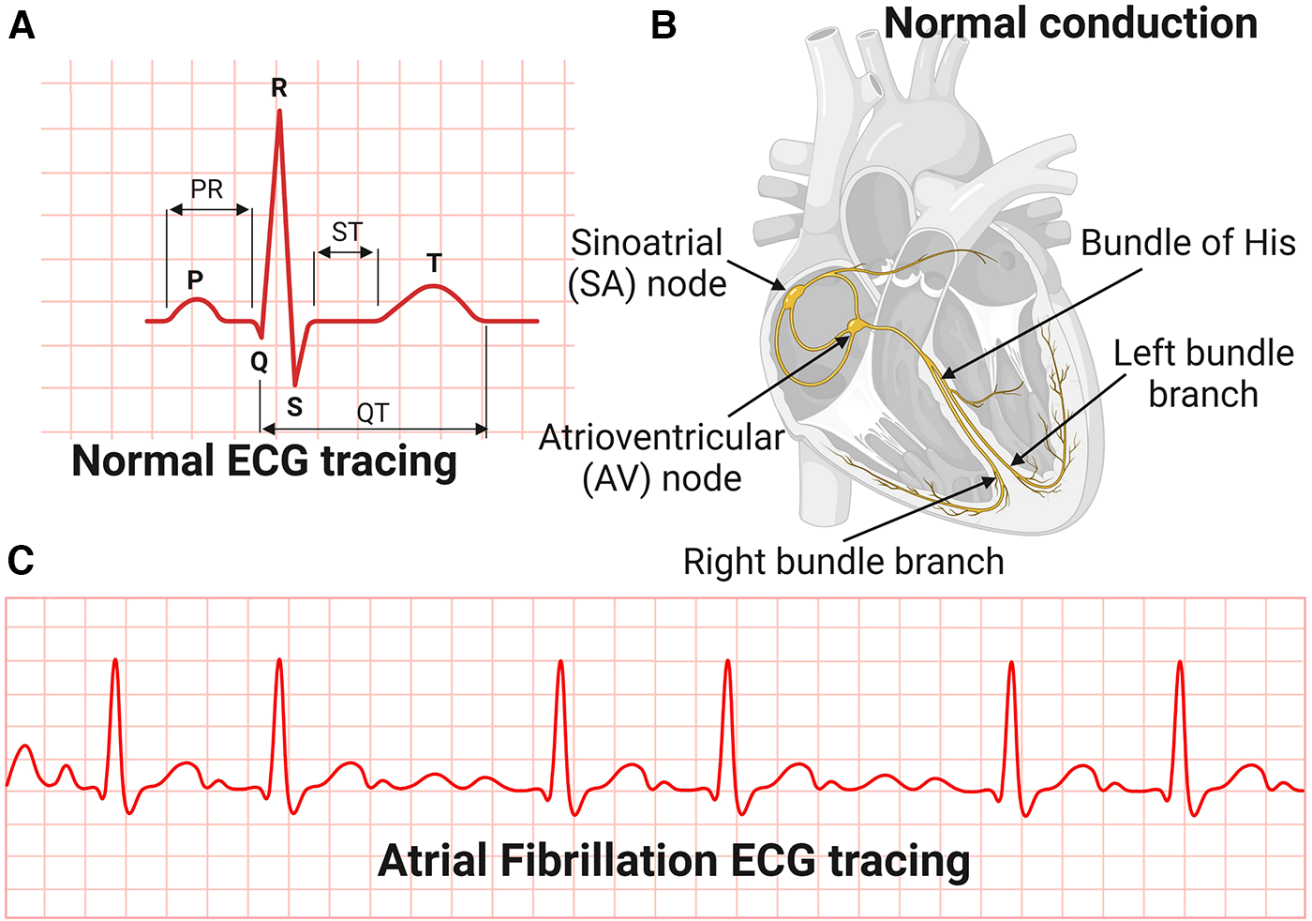
Electrocardiography and conduction system of the heart. (**A**) Normal Electrocardiography (ECG). (**B**) Conduction system of the heart. (**C**) Atrial Fibrillation ECG tracing.

The most common manifestation or complication of hypertensive heart disease is cardiac arrhythmias, and the most common among these is AFib (80). AFib is an irregular and very rapid heart rhythm associated with increased risk for blood clots in the heart, stroke, and heart failure (81–84). AFib can be detected on an electrocardiography (ECG) (Figure 3). During normal heart conduction, electrical signals from the sinoatrial node travel through the atria to the atrioventricular node, passes through the ventricles causing them to contract (Figures 3A,B). In AFib, the electrical signals conduct in a chaotic manner firing from multiple locations leading to faster and irregular heartbeats and characterized by lack of a P-wave and irregular QRS complexes on an ECG (85) (Figure 3C).

Although effective control of blood pressure prevents AFib, some antihypertensive drugs such as thiazide diuretics used to control blood pressure can contribute to AFib risk by inducing hypokalaemia and hypomagnesemia (80). The mechanisms associated with AFib are not yet clear but include modulation of L-type Ca^2+^ and K + currents and gap junction function, cardiac structural remodeling and autonomic remodeling which is characterized by altered sympathovagal activity and hyperinnervation (85). One explanation for the chaotic rhythms in AFib is that structural remodeling characterized by atrial fibrosis occurring in hypertensive heart disease is associated with reentry of a self-sustaining cardiac rhythm abnormality (86). Ectopic conduction activities originating from the pulmonary veins is one of the common triggers of AFib due to specific action potential properties of the pulmonary vein cardiomyocytes (86). Overall, the mechanisms of conduction arrhythmias associated with hypertensive heart disease are not yet clear. In addition, sex differences in the risk, pathogenesis and outcomes of AFib also exist but data is limited (87).

### Mechanisms of coronary artery disease associated with hypertensive heart disease

**Figure 4:**
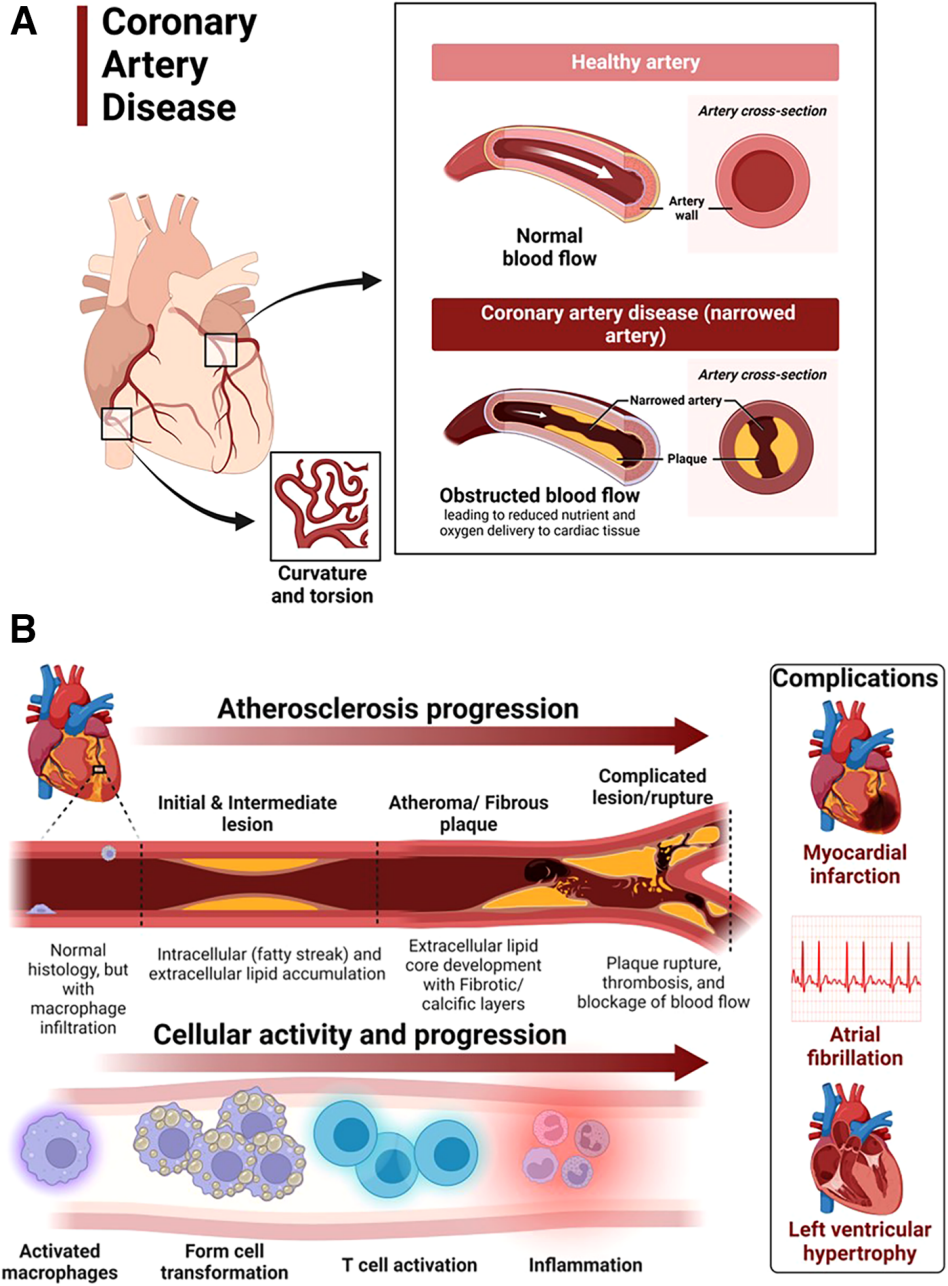
Atherosclerosis progression in coronary artery disease. (**A**) Coronary artery disease is characterized by narrowed coronary arteries due to advanced atherosclerotic fatty plaque. (**B**) Atherosclerosis in coronary artery disease and complications. Atherosclerosis is initiated by fatty streak and macrophage infiltration to rid of cholesterol deposits in the coronary arteries. Macrophages that have engulfed cholesterol deposits transform into lipid laden foam cells, enter the subendothelial layer and also activate the immune system leading to inflammation. Increased deposition of fat, calcium and persistent inflammation leads to formation of fibrotic and calcic changes that can result in plaque rupture, thrombosis and blockage of blood flow in the vasculature. The resulting complications of advanced atherosclerotic lesions include myocardial infarction, atrial fibrillation and left ventricular hypertrophy among others. Activated macrophages and T cells are the main players.

Coronary artery disease in hypertensive heart disease is accelerated by chronic elevation of blood pressure that induces endothelial dysfunction and exacerbates atherosclerotic processes (88). LVH exacerbates coronary artery disease by promoting myocardial ischemia mediated by a decreased coronary reserve and increased myocardial oxygen demand (88). Atherosclerosis remains the main cause of cardiovascular diseases and hypertensive heart disease accelerates complications of atherosclerotic diseases (89). Coronary arteries are considered the most susceptible blood vessels to atherosclerosis in the entire cardiovascular system due to their structurally higher curvature and torsion that plays a role in the localization of early coronary artery thickening (90) (Figure 4A). Blood pressure disturbances and irregularities and cardiac remodeling associated with hypertensive heart disease increase the risk of coronary artery disease and related complications such as myocardial infarction, angina, heart failure and AFib (88, 91, 92) (Figure 4B). The risk factors for coronary artery disease are similar with those associated with hypertension (93, 94). Sex differences in the burden, pathogenesis or severity of coronary artery disease do exist. For example incidental finding of coronary microvascular dysfunction is more common in women than men (95–98). In addition, coronary microvascular dysfunction occurs more in women than in men (99).

The mechanisms underlying the pathophysiology of coronary artery disease include fatty streak formation that activates macrophages to take up these lipids and deposit in the sub endothelium (100, 101). Immune cells including T cells are activated and recruited, secreting inflammatory cytokines in the process that results in the deposition of oxidized low-density lipoprotein (LDL) particles and collagen to form a stable subendothelial plaque that grows with time predisposing to vessel occlusion and atherothrombotic activity in chronic hypertension (101, 102) (Figure 4B). The immune system is intricately involved in atherosclerotic processes. Studies using apolipoprotein E–deficient mice have reported that when chronic inflammation does not resolve, tertiary lymphoid organs emerge in tissues and the adventitia of the aorta which become infiltrated with activated dendritic cells, B cells, and T cells of varying types (103, 104). These cells seem to target unknown antigens released from injured tissue and contribute to advanced atherosclerosis. The whole mark of disease progression is mediated by autoimmune B and T cells that become overly activated as a result of failure of anti-inflammatory effects to remove the continuous discharge of antigens from injured atherosclerotic tissue (104). Further, evidence of formation of neuroimmune cardiovascular interfaces characterized by expanded axon networks and activated artery-brain circuit activity in the adventitia is another proposed mechanism contributing to the progression of atherosclerosis in coronary artery disease (105). The whole process of atherosclerosis development has been described elsewhere (106). Shear stress associated with hypertensive heart disease and atherosclerotic changes leading to plaque progression and remodeling activates PKC epsilon, c-Jun N-terminal Kinase (JNK) MAP kinase, and p53 that worsen endothelial remodeling in the vasculature (107). High shear stress also activates matrix metalloproteinases (MMPs) resulting in thinning of artery wall and eccentric remodeling (107).

Although there are many proposed models of atherosclerosis based on animal studies and a few focused on humans, the challenge remains in translating our understanding to clinical practice (108).

## Dietary salt in hypertensive heart disease and its complications

Excess dietary salt is associated with the development of hypertension and increases the risk for cardiovascular disease, stroke and death (109, 110). Through several mechanisms, excess dietary salt modulates endothelial function and structure, increases systemic peripheral resistance, modulates nervous system function and activates cells of the immune system (109, 111, 112) and accelerates the complications of hypertensive heart disease. The adverse effects of salt also affect normotensive individuals (113–115). Reduction in salt intake of less than 5 grams per day has been shown to lower the risk of developing hypertension and ameliorate cardiovascular diseases (110, 116–125). However, programs aimed at reducing salt intake at population level face a lot of compliance challenges (126).

### Salt sensitivity of blood pressure

Although excess dietary salt raises blood pressure, the effect of salt on blood pressure is variable in the population (127). While significantly elevating blood pressure in some individuals (salt sensitivity), excess salt has no effect on blood pressure in others (salt resistant) and there is a group of individuals (about 15%) whose blood pressure increases with low salt intake (inverse salt sensitivity) (127–129). Salt sensitivity of blood pressure (SSBP) results in part from genetic polymorphisms in genes regulating sodium handling and those not related to sodium handling such as the Protein Kinase CGMP-Dependent 1 (PRKG1), cytochrome b-245 alpha (CYBA) chain (also known as p22-phox), branched chain amino acid transaminase 1 (BCAT1), Solute Carrier Family 8 Member A1 (SLC8A1), SLC4A5, Angiotensin II Receptor Type 1 (AGTR1), Selectin E (SELE), cytochrome P450 family 4 subfamily A member 11 (CYP4A11), Neuronal precursor cell expressed developmentally down-regulated 4-like (NEDD4l) and Visinin Like 1 (VSNL1) (129–133). As explained above, RAAS activation leads to vasoconstriction, increased systemic vascular resistance (SVR) and elevation in blood pressure (134). In individuals with SSBP, RAAS is altered in that renin stimulation is reduced in salt depletion and the mechanisms are not adequate to suppress renin in high salt intake hence worsening the adverse effects of salt on blood pressure (135–138).

### Salt induced immune-activation in the skin in hypertensive heart disease

The handling of salt by the kidney and how salt contributes to water retention and elevated blood pressure is well known. The current dogma that sodium in the interstitial space equilibrates with plasma has been challenged in emerging studies that have now identified extrarenal handling of sodium that contributes to hypertension and sustenance of blood pressure in hypertensive heart disease (139, 140). It is now known that sodium can accumulate in tissues and skin without commensurate volume retention and activate innate and adaptive immunity leading to or sustaining hypertension (139, 141). Accumulation of salt in the skin is associated with autoimmune disease severity and heightening of inflammation in several diseases such as lipedema, diffuse cutaneous systemic sclerosis, multiple sclerosis, psoriasis and systemic lupus erythematosus (142–146). Several studies have demonstrated similar findings of increased sodium accumulation in the skin in hypertension using sodium magnetic resonance imaging (^23^Na MRI) (147–150). For dietary sodium to reach the skin from the intestinal lumen, it is first absorbed across the apical membrane of enterocytes through sodium-hydrogen exchangers (NHE), sodium glucose cotransporter 1 (SGLT1), sodium-dependent phosphate transporter 2b (NaPi2b), glucose transporters (GLUT) and endothelial sodium channels (ENaC) and pumped across the basal membrane of the intestine into the interstitium by Na-K ATPases (151–154). From the insterstitium sodium diffuses into the intestinal capillaries for transport. In the vasculature, excess dietary salt diminishes the buffering capacity of the negatively charged glycocalyx lining the endothelium and the red blood cells leading to extravasation of sodium and accumulation of salt in the interstitial tissues (140, 155, 156). Accumulation of salt in the skin increases the density and hyperplasia of the lymph-capillary network and this effect is mediated by activation of tonicity-responsive enhancer binding protein (TonEBP) in mononuclear phagocyte system (MPS) cells (140). TonEBP binds to and activates the promoter of the gene encoding vascular endothelial growth factor-C (VEGF-C) resulting in VEGF-C secretion and trapping by macrophages, augmenting interstitial hypertonic volume retention, decreasing endothelial nitric oxide synthase expression and elevating blood pressure in response to excess dietary salt (140). In addition, the hypertonic milieu contributed by the accumulation of sodium in the skin that induces the expression of VEGF-C increases lymphangiogenesis as a compensatory mechanism to eliminate sodium from the skin but this process is usually disrupted in hypertension, exacerbating hypertensive heart disease (147, 148). Low salt diet has shown to improve dermal capillary density and blood pressure in hypertension (157).

A group by Laffer et al. investigated hemodynamic changes in individuals with SSBP and found that compared to salt resistant individuals, individuals with SSBP had higher total peripheral resistance after salt loading which did not change after salt depletion and further, they also gained weight during salt loading but lost more weight during salt depletion that reflected failure to correct fluid retention (158). This study suggests that individuals with SSBP are unable to maintain and modulate a proper hemodynamic balance that reflects a dysfunction in the storage of salt in the interstitial compartment probably due to vascular dysfunction (156, 159).

**Figure 5:**
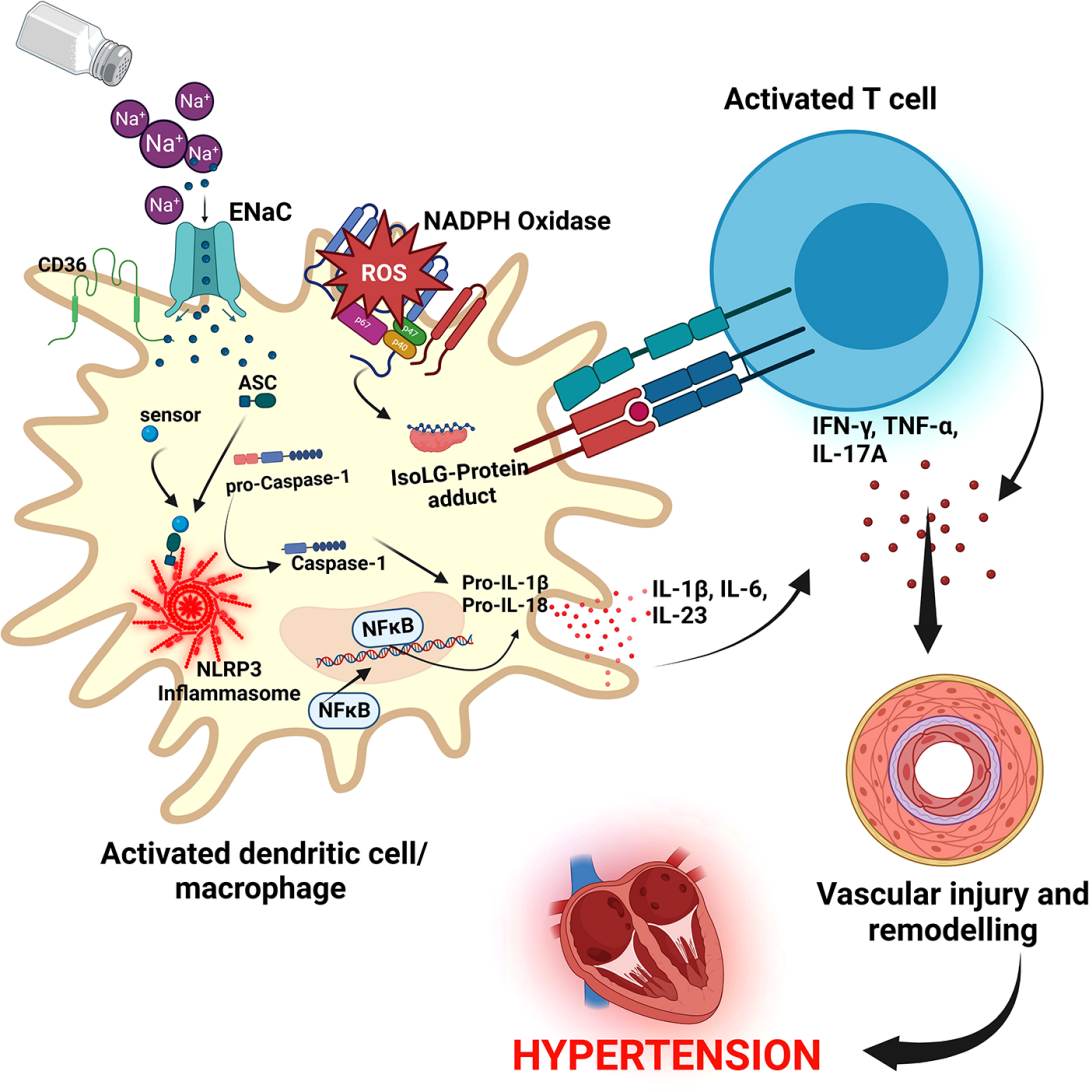
Salt induced hypertension. In high salt environments, dendritic cells or macrophages become activated through increased intracellular sodium that enter *via* the epithelial sodium channel (ENaC). Sodium activates NADPH oxidase and the inflammasome leading to formation of Isolevuglandins(IsoLGs)-protein adducts that are processed in major histocompatibility molecules and presented to T cells, activating them. Activated T cells produce inflammatory cytokines IFN-*γ*, TNF-α and IL-17A that lead to hypertension. NF-Kb, Nuclear factor kappa-light-chain-enhancer of activated B cells; IFN-*γ*, interferon gamma; TNF-α, Tumor necrosis factor alpha; ROS, reactive oxygen species; NLRP3, NACHT, LRR, and PYD domains-containing protein 3; ASC, Apoptosis-associated speck-like protein containing a CARD.

Resident macrophages and dendritic cells in the interstitium of the skin are activated in the presence of excess dietary salt and via increased activity of the ROS producing reduced nicotinamide adenine dinucleotide phosphate (NADPH)-oxidase, the ROS oxidize arachidonic acid leading to formation of Isolevuglandins (IsoLGs) (160, 161). IsoLGs adduct to lysine residues and alter intracellular protein structure and function and the resulting IsoLG-protein adducts act as neoantigens presented to and activating T cells (160). The activated T cells produce interferon-gamma (IFN-*γ*), interleukin 17A (IL-17A) and tumor necrosis factor-alpha (TNF-α) which causes vascular damage and lead to hypertension (160, 162). The activated macrophages and dendritic cells produce inflammatory cytokines IL-1β, IL-6 and IL-23 which induce T cell proliferation and production of inflammatory cytokines implicated in hypertension (160) (Figure 5). It has been demonstrated in many studies that T cells infiltrate the kidney causing vascular injury via inflammatory cytokines and increased oxidative stress and contributing to salt sensitive hypertension (163–165).

### Cellular pathways activated by excess dietary salt

Several cellular pathways have been implicated in salt sensitive hypertension. The NACHT, LRR, and PYD domains-containing protein 3 (NLRP3) inflammasome is an oligomeric complex containing the NOD-like receptor NLRP3, the adaptor Apoptosis-associated speck-like protein containing a caspase recruitment domain (ASC), and caspase-1 implicated in salt sensitive hypertension (166). The inflammasome is activated when NF-*κ*B upregulates the inflammasome components and pro-IL-1β leading to the assembly of components to form the NLRP3 inflammasome signaling complex (134, 166) (Figure 5). Activation of the NLRP3 inflammasome leads to the release of pro-inflammatory cytokines IL-1β and IL-18 via pyroptosis that involves the cleavage of gasdermin D and development of pores in the membrane of cells through which the cytokines and other cellular contents are released (134, 166). It has been demonstrated that the NLRP3 inflammasome can be activated in high salt environments in an ENaC-dependent manner leading to IsoLG-protein adduct formation in dendritic cells and macrophages and antigen presentation to activate cells of the adaptive immune system and leading to hypertension as explained above (167).

In high salt diets, small guanosine triphosphatases (GTP)ases Rho and Rac kinases are activated and lead to activation of sympathetic nerve outflow that results in blood pressure elevation (168). In vascular smooth muscles, Rho kinases facilitate vasoconstriction though GPCRs and Wnt pathways, and in vascular endothelium, Rho/Rho kinase inhibits nitric oxide (NO) leading to increased vascular resistance and vascular tone and salt sensitive hypertension (168).

A study by Chu et al., in 329 subjects looked at growth factors that are produced in relation to the activation of the phosphoinositide 3-kinase/ Ak strain transforming (PI3K-Akt) pathway, which is activated through serine and/or threonine phosphorylation of a range of downstream substrates (169). They found that individuals with SSBP had elevated levels of several growth factors compared to salt resistant group (169). The signal transduction PI3K-Akt Pathway regulates metabolism, proliferation, cell survival, growth and angiogenesis (170). The PI3K-Akt Pathway activation has been implicated to contribute to the progression of atherosclerotic plaque formation and pathological changes in the vasculature leading to hypertension and cardiovascular disorders in many studies (171–175). Several other cellular pathways associated with salt sensitive hypertension such as the WNK signaling pathway (176), Kelch-like 3/Cullin 3 ubiquitin ligase complex (177), brain Gαi2-proteins of GPCR (178, 179) in the central nervous system, MAPK/extracellular signal regulated kinase [ERK] mediated by angiotensin II in vascular smooth muscles (180), and redox signaling (181) have been well elucidated.

## Genetic predisposition in hypertensive heart disease complications

There is substantial evidence for genetic involvement in hypertensive heart disease and its complications (heart failure, AFib, and coronary artery disease). Evidence from observational, sibling and longitudinal twin studies reported that LVH phenotypes are highly heritable (182–184). Specific variants have been associated with abnormalities in cardiac structure and function related to hypertensive heart disease using gene association and genome-wide association studies (185, 186). Genome wide association studies and international collaborative metanalysis studies have also reported more than 30 gene loci associated with AFib (187–191). Several studies have demonstrated that most polymorphisms associated with blood pressure also increases the risk for coronary artery disease (192), incident hypertension and cardiovascular diseases (193). We found several studies that have reported genes associated with cardiomyopathies and heart failure such as myosin heavy chain 7 (MYH7), troponin T (TNNT2), troponin I (TNNI3), cardiac myosin binding protein 3 (MYBPC3), tropomyosin alpha-1 (TPM1), Lamin A/C (LMNA/C), plakophilin 2 (PKP2), desmocollin 2 (DSC2), desmoglein 2 (DSG2), desmoplakin (DSP), plakoglobin (JUP) and titin (194). We also know that the genetic component requires interaction with environmental factors for the effect or risk for hypertension and cardiovascular disease to be heightened (195). The genetic predisposition to hypertensive heart disease has been extensively reviewed and studied elsewhere (185, 192, 195–200).

## Current diagnostic techniques

Echocardiography, carotid ultrasound and cardiac magnetic resonance imaging are important diagnostic techniques used in the clinic to detect functional and structural changes in the heart such as occurs in LVH (1, 50, 201). Echocardiography is cheap, readily available and more preferred to the high cost and limited availability of the gold standard, cardiac magnetic resonance imaging (42). Cardiac magnetic resonance imaging is a noninvasive, tomographic, nonionizing technique used to detect structural changes in the heart and therefore important for the diagnosis of hypertrophic cardiomyopathy, coronary heart disease, congenital heart disease, heart failure and other cardiac abnormalities (202).

## Therapy for hypertensive heart disease and its complications

Controlling hypertension with current medication reduces the risk for complications and adverse cardiovascular events. The current US and European guidelines have extensively discussed therapy for hypertension and all related cardiovascular complications (2, 203). Thus, we will briefly focus on recent clinical studies reporting potential therapies that are especially used in combination for the treatment of LVH, AFib, coronary artery disease, and heart failure.

### Left ventricular hypertrophy therapies

Patients with LVH benefit remarkably from intensive blood pressure lowering (<120 mmHg) to prevent complications (36). In clinical trials, several therapies have been reported to reduce LVH and its complications. Use of the neprilysin inhibitor sacubitril used for treatment of heart failure and the angiotensin receptor blocker valsartan was associated with reduced left ventricular mass index when compared to the angiotensin receptor blocker (ARB) Olmesartan, in participants with hypertension (204). Another clinical trial reported that combination of the ARB telmisartan and simvastatin did not only significantly reduce blood pressure but was able to reverse LVH and improve left ventricular systolic function (205). Similar findings were reported for a triple fixed dose combination of perindopril/indapamide/amlodipine (angiotensin-converting enzyme inhibitor (ACEI)/diuretic/calcium channel blocker (CCB)) in patients with essential hypertension followed for 14 months (206). Another interesting finding is from a clinical trial by Lal et al. where they used allopurinol, a xanthine oxidase inhibitor commonly used to reduce plasma uric acid in patients with gout, to determine its efficacy in reducing LVH (207). High dose allopurinol was more effective in reducing left ventricular mass and LVH when compared to febuxostatin (207) but caution must be exercised in using allopurinol in normouricemic individuals with controlled blood pressure as it can increase oxidative stress (208). In general, it appears that significant reversal of LVH is greater when both RAAS and sympathetic nervous system (SNS) inhibitors are used compared to drugs that just target blood pressure reduction (209). Other drugs as well as natural compounds or interventions used in combination have also been reported in clinical trials to ameliorate progression of LVH, examples include a nutraceutical combination of berberine, red yeast rice extract and policosanol (210), azelnidipine (211), losartan (212), low-dose eplerenone (213), metformin in patients with coronary artery disease without diabetes (214), diets low in fat and carbohydrate and regular consumption of green tea (215, 216).

### Therapies for atrial fibrillation

Several clinical trials have reported beneficial therapies in the management of AFib. A few are discussed below.

When AFib is controlled, patients remain at risk for cardiovascular events, however, early rhythm control achieved by using antiarrhythmic drugs or atrial fibrillation ablation was effective in treating AFib and reducing the risk for cardiovascular events (217). In clinical practice, patients are first prescribed drugs such as beta blockers or a CCB in patients with asymptomatic AFib but a few clinical trials found that cryoballoon ablation was more effective compared to drug therapy as initial therapy for AFib (218, 219). Thus, rhythm control may be beneficial in both asymptomatic and symptomatic AFib (220). Further, radiofrequency ablation may delay or prevent paroxysmal AFib from progressing into persistent AFib (221). Despite its beneficial effect, caution should be exercised, as catheter ablation may also increase left atrial stiffness and worsen post-ablation diastolic function (222). Additional interventions for the management of AFib and its complications have been reported in other clinical trials elsewhere (223–227).

### Therapies for coronary artery disease and heart failure

Patients with coronary artery disease also benefit from several interventional strategies including dietary interventions (228, 229), rivaroxaban monotherapy and other drugs (230–232), and physical exercise (233, 234). Further, lifestyle modifications have also been reported to be beneficial (235).

To alleviate heart failure and reduce its complications, several interventions are available (236). For example, in a clinical trial by Hieda et al. they found that physical exercise training for one year reversed left ventricular myocardial stiffness in patients with stage B heart failure with preserved ejection fraction that is characterized by LVH and N-terminal pro-B-type natriuretic peptide or high-sensitivity troponin (237). Therapeutic interventions for patients with heart failure also exist. Empagliflozin, dapagliflozin and spironolactone improves and ameliorates adverse outcomes of heart failure with persevered ejection fraction (238–240). In addition, individualized nutritional support as well as treatment with vericiguat for hospitalized patients with heart failure is also beneficial in reducing the risk for death and morbidity (241–243). Further, in patients with acute decompensated heart failure, usage of levosimendan in combination with Shenfu injection was effective in improving hemodynamics and enhance myocardial contractility (244). In severe heart failure where therapy is limited, use of omecamtiv mecarbil therapy is reported to have beneficial effects in reducing adverse outcomes (245). Management of heart failure is discussed in detail in the US and European guidelines.

## Future directions

Future studies should focus on clinical studies (especially prospective) to understand the pathogenesis and complications of hypertensive heart disease as there are few studies in this area. Understanding the implications of physiological and pathological LVH and the potential for regression will be important for clinical application.

## Conclusions

Hypertensive heart disease progresses through several mechanisms that amplify and increase the risk for adverse complications. Excess dietary salt is one of the modifiable factors that contribute enormously to the pathogenesis of hypertensive heart disease. Reduction of dietary salt has potential to reduce blood pressure and the risk for development of hypertensive heart disease.
